# P18 Audit on compliance with the preventive measures on *Clostridioides difficile*

**DOI:** 10.1093/jacamr/dlac133.022

**Published:** 2023-01-19

**Authors:** I Marques Reis, V Vikneswaramoorthy, G McHale, M Mullahy, J Valiakalayil, T Hallinan, M Cruz, L Nieto-Aponte, S Sibartie

**Affiliations:** Mayo University Hospital, Ireland; Mayo University Hospital, Ireland; Mayo University Hospital, Ireland; Mayo University Hospital, Ireland; Mayo University Hospital, Ireland; Mayo University Hospital, Ireland; Mayo University Hospital, Ireland; Mayo University Hospital, Ireland; Mayo University Hospital, Ireland

## Abstract

**Background:**

*Clostridioides (Clostridium) difficile* persists to be a significant pathogen for healthcare acquired infections. Antibiotic usage is the main cause for the overgrowth of this organism and resulting in *C. difficile* infections (CDI). Other factors that are known to contribute are usage of proton pump inhibitors, laxatives usage and poor compliance with infection prevention control guidance in health care settings. The audit was carried out to analyse if the local guidance is followed on recognition of new onset of diarrhoea in an inpatient.

**Objectives:**

Analyse compliance rate with our local guidance in Mayo University Hospital to be followed when inpatient has a new onset of diarrhoea. This to include reviewing of medications; such as laxatives, proton pump inhibitor (PPI) and antibiotics; prompt isolation and CDI precautions were commenced, samples sent for testing and commencing stool chart.

**Methods:**

The data of patients was collected by reviewing the chart and collected data was stored and analysed using excel spreadsheet.

**Results:**

The audit was carried out over a 4 month period from 10.06.22 until 14.10.22 in 7 wards. 31 patients were recorded as having diarrhoea with 69% (n=19) female and 39% (n=12) males during their inpatient stay. Majority of the recorded patients, which is 29% (n=9) were in medical male ward. The age of the patients ranged from 24 to 92 years of age with a median of 68 years. The median number of days from the admission until onset of diarrhoea was 5. On identifying new onset of diarrhoea, 32% (n=10) had recorded documentation of medications review. Out of 31 patients, 90% (n=28) were on PPI and only 21% (n=6) of them were held, while 7% (n=2) of them had their medication reviewed and was advised to continue the PPI as for clinical indication. Ninety percent (n=28) of patients were on regular laxatives and after the onset of diarrhoea only 39% (n=11) had the laxatives held. Of all the recorded patients, 32% (n=10) of patients had documentation of medication review and 19% (n=6) were continued on antibiotics for organisms other than CDI after discussion with clinical microbiology team. Fifty-five percent (n=17) of patients were isolated after the onset of new diarrhoea while the rest remained in a shared bay as per the discussion with clinical teams, while awaiting the stool results. Sixty-five percent (n=20) had a stool culture sent for CDI testing after the onset of new diarrhoea. Of these patients, 25% (n=5) tested positive for *C. difficile.*

**Conclusions:**

Compliance to local guidance should be followed more strictly to avoid development of hospital acquired CDI and to prevent outbreaks in the healthcare setting.

